# Prevalence and Risk Factors of Type 2 Diabetes Mellitus among Depression Inpatients from 2005 to 2018 in Beijing, China

**DOI:** 10.34133/hds.0111

**Published:** 2025-03-05

**Authors:** Peng Gao, Fude Yang, Qiuyue Ma, Botao Ma, Wenzhan Jing, Jue Liu, Moning Guo, Juan Li, Zhiren Wang, Min Liu

**Affiliations:** ^1^ Department of Epidemiology and Biostatistics, School of Public Health, Peking University, Beijing, China.; ^2^ Beijing Huilongguan Hospital, Peking University Huilonguan Clinical Medical School, Beijing, China.; ^3^ Department of Hernia and Abdominal Wall Surgery, Beijing Chaoyang Hospital, Capital Medical University, Beijing, China.; ^4^ Vanke School of Public Health, Tsinghua University, Beijing, China.; ^5^ Beijing Municipal Commission of Health and Family Planning Information Center, Beijing Municipal Commission of Health and Family Planning Policy Research Center, Beijing, China.; ^6^ Beijing Geriatric Hospital, Beijing, China.

## Abstract

**Background:** There are few data on the comorbidity of diabetes in Chinese patients with depression. We aimed to calculate the prevalence and explore risk factors of type 2 diabetes mellitus (T2DM) among depression inpatients from 2005 to 2018 in Beijing. **Methods:** This study is a cross-sectional study. The data collected from 19 specialized psychiatric hospitals in Beijing were analyzed. The prevalence of T2DM and its distribution were analyzed. The multivariable logistic regression was performed to explore the risk factors of T2DM. **Results:** A total of 20,899 depression inpatients were included. The prevalence of T2DM was 9.13% [95% confidence interval (CI), 8.74% to 9.52%]. The prevalence of T2DM showed an upward trend with year (*P* for trend < 0.001) and age (*P* for trend < 0.001). The prevalence of T2DM was higher among readmitted patients (12.97%) and patients with comorbid hypertension (26.16%), hyperlipidemia (21.28%), and nonalcoholic fatty liver disease (NAFLD) (18.85%). The prevalence of T2DM in females was lower than in males among patients aged 18 to 59 years, while the prevalence of T2DM in females was higher than in males among patients aged ≥60 years. T2DM was associated with older age [adjusted odds ratios (aORs) ranged from 3.68 to 29.95, *P* < 0.001], hypertension (aOR, 3.01; 95% CI, 2.70 to 3.35; *P* < 0.001), hyperlipidemia (aOR, 1.69; 95% CI, 1.50 to 1.91; *P* < 0.001), and NAFLD (aOR, 1.58; 95% CI, 1.37 to 1.82; *P* < 0.001). **Conclusions:** The prevalence of T2DM among depression inpatients from 2005 to 2018 in Beijing was high and increased with the year. Depression inpatients who were older and with hypertension, hyperlipidemia, and NAFLD had a higher prevalence and risk of T2DM.

## Introduction

Depression, also known as depressive disorder, is characterized by depressed mood, decreased interest, and anhedonia. In severe cases, individuals may exhibit psychotic symptoms such as hallucinations or delusions [1]. According to the World Health Organization (WHO), depression is a common mental disorder and about 5% of adults suffer from it [2]. The China Mental Health Survey conducted from 2013 to 2015 indicated that the lifetime prevalence of depression among adults in mainland China was 6.8% [3]. Diabetes mellitus is a chronic disease characterized by persistent hyperglycemia, which leads to serious damage to many of the body’s systems over time. Type 2 diabetes mellitus (T2DM) accounts for more than 95% of all patients with diabetes, and almost all T2DM patients are adults [4]. The China Chronic Disease and Risk Factors Surveillance conducted by the Chinese Center for Disease Control and Prevention indicated that the prevalence of diabetes among adults in mainland China increased from 10.9% in 2013 to 12.4% in 2018 [5]. The Global Burden of Disease study ranked disability-adjusted life-years (DALYs) due to 369 diseases and injuries and found that the DALYs caused by depression increased from the 19th in 1990 to the 13th in 2019, and the DALYs caused by diabetes increased from the 20th in 1990 to the 8th in 2019 [6]. Both depression and diabetes have caused heavy disease burdens and led to serious public health problems.

The comorbidity of depression and diabetes has attracted the attention of many researchers. There seems to be a bidirectional relationship between depression and diabetes. A meta-analysis of longitudinal studies showed that the risk of developing diabetes was 38% higher in depressed subjects compared with nondepressed subjects [7]. Another meta-analysis showed that the risk of developing depression was 28% higher in patients with diabetes compared with subjects without diabetes [8]. However, a review indicated that the evidence for a direct causal association between depression and T2DM was insufficient but doctors should be aware of the co-occurrence of these 2 diseases [9]. The interaction of depression and diabetes seriously damages the health of patients and has a negative influence on patients’ quality of life [10]. A meta-analysis found that depression was associated with diabetes complications, such as diabetic neuropathy, retinopathy, nephropathy, macrovascular complications, and sexual dysfunction [11]. Higher levels of depressive symptoms among patients with diabetes reduce adherence to a diabetic diet and the ability to take hypoglycemic medications correctly [12]. Among patients with T2DM, compared with the nondepressed group, the mortality risk increased by 46% in the minor depression group and increased by 43% in the major depression group [13]. In addition, the combination of chronic diseases, such as depression and diabetes, leads to a heavy financial burden for both the social healthcare system and patients (more frequent physician visits, more hospital bed-days, more medication use, and higher out-of-pocket expenses) [14].

A review pointed out that most of the studies explored the relationship between depression and diabetes based on depressive symptom scales, not clinical diagnosis [9]. Many studies were conducted in patients with diabetes rather than depression. These bring difficulty in understanding the prevalence of diabetes in patients with depression. Moreover, less data are available for the Chinese population. In English publications, the recent umbrella review (published in 2021) summarized 3 systematic reviews to pool the prevalence of T2DM in patients with depression [15]. Among the 3 systematic reviews, only 2 original studies were conducted in Taiwan (a province of China). These 2 studies reported that the prevalence of T2DM in major depressive disorder was 11.6% [16] and 6.0% [17], respectively. In Chinese publications, only a few studies from a single hospital have reported the prevalence of diabetes in patients with depression.

We have previously analyzed the prevalence and T2DM among psychiatric inpatients in Beijing but have not analyzed those among patients with depression [18]. Depression is one of the most common mental disorders and accounted for the largest proportion (37.3%) of DALYs in the overall mental disorders in 2019 [19]. Patients with depression are the key population to improving the damage by of patients with mental disorders due to chronic diseases. It is necessary to analyze the comorbidity of chronic diseases, especially diabetes, among patients with depression. In this study, we aimed to analyze the prevalence and risk factors of T2DM among depression inpatients from 2005 to 2018 in Beijing to provide scientific evidence for strategies to improve chronic comorbidities in depressed populations.

## Methods

### Study design and data source

This study is a cross-sectional study. The data source was the medical record front page data of 19 specialized psychiatric hospitals in Beijing, which was collected by the Beijing Municipal Commission of Health and Family Planning. The data contain all psychiatric inpatients hospitalized from 2005 January 1 to 2018 December 31.

### Criteria of inclusion and exclusion

1. Inclusion criteria: (a) Depression inpatients; (b) adults (≥18 years old).
2. Exclusion criteria: In the same calendar year, if a patient had more than one record, the last record was retained and others were excluded.

### Definitions of variables and outcome

The variables analyzed in this study were as follows: (a) demographic information: sex, age, ethnicity, marital status, occupation, and medical insurance; (b) hospitalization information: hospital level, frequency of hospitalization, and length of hospitalization; (c) disease information: type of depression, T2DM, hypertension, hyperlipidemia, and NAFLD; (d) medication information: lithium carbonate, antipsychotic drug, and nootropic drug.

Demographic information, hospital level, and length of hospitalization were directly obtained from the medical record front page data. Hospitals in China are designated as primary, secondary, or tertiary institutions [20]. Hospitals with higher levels have more beds and provide more comprehensive medical services. For the frequency of hospitalization, a patient whose record showed 1 hospitalization was defined as first-admitted, and a patient whose record showed ≥2 hospitalizations was defined as readmitted. The use of medications can also be directly found in the records. Disease diagnosis was based on the International Classification of Diseases 10th Revision (ICD-10) [21]. A patient was defined as having the corresponding disease if the corresponding codes were found in the “main diagnosis” or “other diagnosis” on the medical record front page. The 5 diseases and diagnosis codes involved in this study were as follows:

1. Depression: F32 (depressive episode), F33 (recurrent depressive disorder), and F34.1 (dysthymia);
2. T2DM: E11 (type 2 diabetes mellitus), E14 (unspecified diabetes mellitus);
3. Hypertension: I10 (essential hypertension), I11 (hypertensive heart disease), I12 (hypertensive renal disease), I13 (hypertensive heart and renal disease), I15 (secondary hypertension);
4. Hyperlipidemia: E78.0 (pure hypercholesterolemia), E78.1 (pure hyperglyceridemia), E78.2 (mixed hyperlipidemia), E78.3 (hyperchylomicronemia), E78.4 (other hyperlipidemia), E78.5 (hyperlipidemia, unspecified);
5. Nonalcoholic fatty liver disease (NAFLD): K76.0 (fatty liver, not elsewhere classified).

The outcome of this study was the prevalence of T2DM among depression inpatients. All the prevalence of T2DM was calculated as shown in the formula below, and the statistical scope was depression inpatients from 2005 to 2018 (except for the prevalence of T2DM by year, whose statistical scope was depression inpatients in the corresponding year).

$$\begin{array}{c} \begin{array}{c} \begin{array}{c} \text{Prevalence of}\;T2\mathrm{DM}=\\ \frac{\text{The number of patients with}\;T2\mathrm{DM}\;\text{from}\;2005\;\text{to}\;2018}{\text{The number of depression inpatients from}\;2005\;\text{to}\;2018}\times 100\% \end{array} \end{array} \end{array}$$ (1)

### Statistical analysis

First, the variables on the medical record front pages were grouped. Second, the prevalence of T2DM was calculated after grouping each variable, and the chi-square test was used to analyze whether there was a difference in the prevalence of T2DM between the subgroups of each variable. Third, multivariate logistic regression analysis was used to calculate each variable’s adjusted odds ratio (aOR) and 95% confidence interval (CI), taking the presence of T2DM as the dependent variable and the remaining variables as the independent variables. In addition, we performed some stratified analyses. Statistical analyses were performed using R (version 4.2.1) and RStudio (version 2023.03.1) software.

## Results

### Characteristics of depression inpatients

**Fig. 1.**
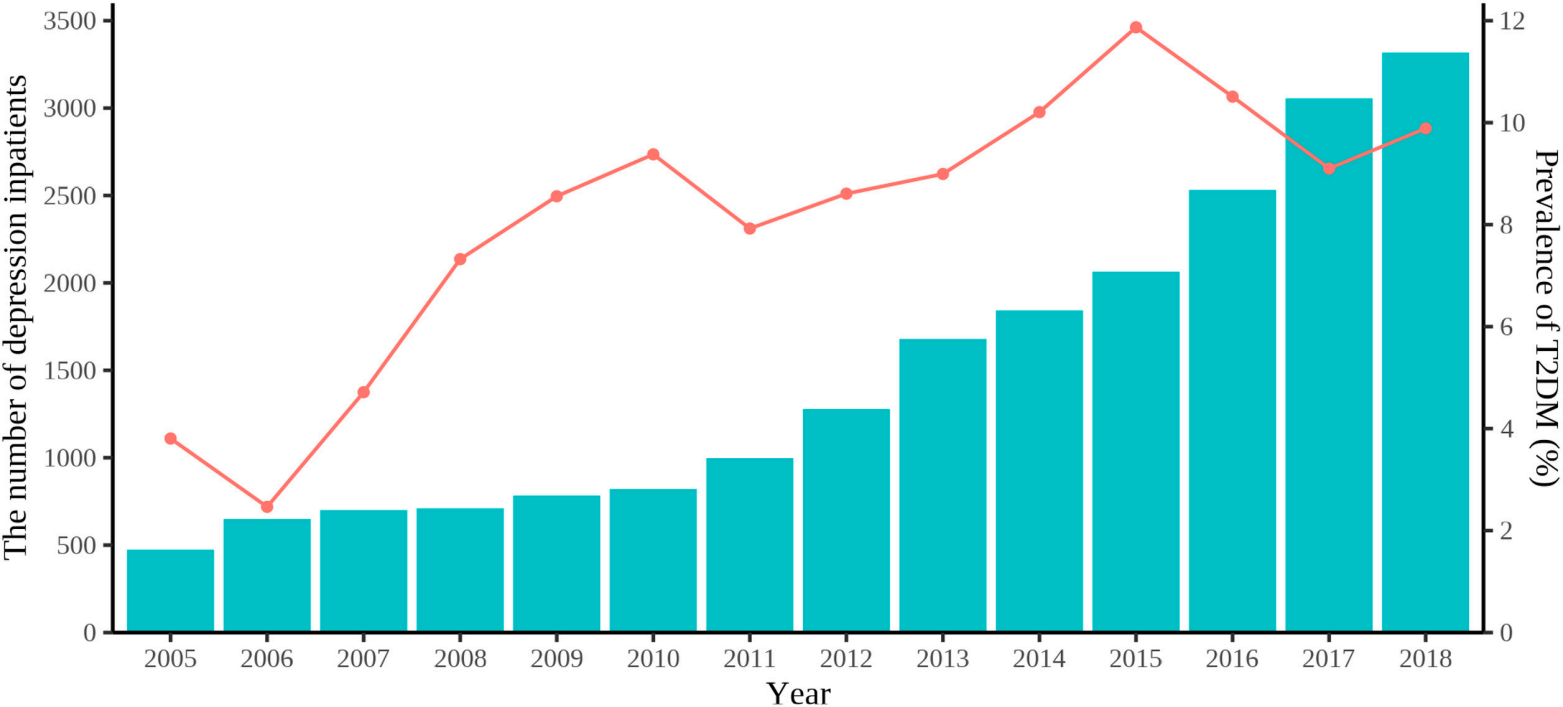
The number of depression inpatients and prevalence of T2DM. The bar graph shows the number of patients, and the line graph shows the prevalence of T2DM.

A total of 20,899 depression inpatients were included for analysis. The number of depression inpatients increased year by year, from 473 in 2005 to 3,317 in 2018 (Fig. 1). As shown in Table, in terms of demographic characteristics, most depression inpatients were female (62.93%), Han Chinese (94.29%), married (69.74%), employed (83.88%), and had medical insurance (73.48%). The proportions of depression inpatients in different age groups ranged from 10.00% in the ≥70 years group to 22.21% in the 50 to 59 years group. In terms of hospitalization status, most depression patients were first-admitted (75.40%) and hospitalized in tertiary hospitals (94.87%). Nearly half of patients were hospitalized for 1 to 30 d (49.23%). The proportion of patients with recurrent depressive disorder (48.29%) and depressive episode (50.32%) was similar, and both accounted for the majority of patients with depression. The proportion of patients with dysthymia was very low (1.39%). In terms of comorbidities, the prevalence of hypertension was the highest (20.05%), followed by hyperlipidemia (12.68%) and NAFLD (9.09%). In terms of medication use, the proportion of those receiving antipsychotic drugs was relatively high (47.73%), while the proportion of those receiving lithium carbonate (2.16%) and nootropic drugs (6.04%) was low.

**Table.** Prevalence and risk factors of T2DM among depression inpatients during 2005–2018. T2DM, type 2 diabetes mellitus; CI, confidence interval; aOR, adjusted odds ratio; NCMS, new rural cooperative medical scheme; UEBMI, urban employee basic medical insurance; URBMI, urban resident medical insurance; NAFLD, nonalcoholic fatty liver disease.

Characteristic	Depression inpatient, *n* (%)	T2DM, *n* (%)	Prevalence of T2DM, % (95% CI)	*P* value for chi-square test	aOR (95% CI)	*P* value for aOR
Sex				0.199		
Male	7,748 (37.07)	681 (35.69)	8.79 (8.17–9.44)		1.00	
Female	13,151 (62.93)	1,227 (64.31)	9.33 (8.84–9.84)		0.92 (0.83–1.03)	0.146
Age (years)				<0.001		
18–29	4,321 (20.68)	17 (0.89)	0.39 (0.23–0.63)		1.00	
30–39	2,919 (13.97)	44 (2.31)	1.51 (1.10–2.02)		3.68 (2.03–6.67)	<0.001
40–49	3,559 (17.03)	186 (9.75)	5.23 (4.52–6.01)		11.21 (6.45–19.47)	<0.001
50–59	4,642 (22.21)	607 (31.81)	13.08 (12.12–14.08)		24.24 (14.04–41.85)	<0.001
60–69	3,368 (16.12)	625 (32.76)	18.56 (17.26–19.91)		29.95 (17.28–51.91)	<0.001
≥70	2,090 (10.00)	429 (22.48)	20.53 (18.81–22.32)		28.80 (16.48–50.34)	<0.001
Ethnicity				0.207		
Han Chinese	19,706 (94.29)	1,812 (94.97)	9.20 (8.80–9.61)		1.00	
Non-Han Chinese	962 (4.60)	82 (4.30)	8.52 (6.84–10.47)		1.27 (0.98–1.63)	0.067
Unknown	231 (1.11)	14 (0.73)	6.06 (3.35–9.96)		0.73 (0.41–1.29)	0.279
Marital status				<0.001		
Married	14,574 (69.74)	1,573 (82.44)	10.79 (10.29–11.31)		1.00	
Unmarried	4,593 (21.98)	72 (3.77)	1.57 (1.23–1.97)		1.11 (0.82–1.49)	0.509
Divorced/widowed/other	1,732 (8.29)	263 (13.78)	15.18 (13.53–16.96)		1.11 (0.95–1.30)	0.206
Occupation				<0.001		
Manager of a government agency, public institution, or enterprise	864 (4.13)	29 (1.52)	3.36 (2.26–4.79)		1.00	
Civil servant/professional/office clerk	1,900 (9.09)	93 (4.87)	4.89 (3.97–5.96)		1.45 (0.93–2.26)	0.098
Worker/farmer	2,038 (9.75)	209 (10.95)	10.26 (8.97–11.65)		1.88 (1.24–2.84)	0.003
Other occupations	12,729 (60.91)	1,087 (56.97)	8.54 (8.06–9.04)		1.97 (1.33–2.92)	<0.001
Retiree	1,726 (8.26)	376 (19.71)	21.78 (19.86–23.81)		1.88 (1.25–2.83)	0.002
Unemployed	1,642 (7.86)	114 (5.97)	6.94 (5.76–8.28)		1.76 (1.13–2.73)	0.012
Medical insurance				<0.001		
NCMS	2,858 (13.68)	167 (8.75)	5.84 (5.01–6.77)		1.00	
UEBMI	7,699 (36.84)	954 (50.00)	12.39 (11.66–13.15)		1.33 (1.10–1.60)	0.004
URBMI	794 (3.80)	111 (5.82)	13.98 (11.64–16.59)		1.19 (0.90–1.58)	0.225
Free medical service	722 (3.45)	50 (2.62)	6.93 (5.18–9.03)		1.19 (0.83–1.69)	0.339
Other insurance	4,005 (19.16)	251 (13.16)	6.27 (5.54–7.06)		1.02 (0.82–1.27)	0.856
Out-of-pocket	4,821 (23.07)	375 (19.65)	7.78 (7.04–8.57)		1.27 (1.04–1.56)	0.022
Hospital level				<0.001		
Tertiary hospital	19,827 (94.87)	1,678 (87.95)	8.46 (8.08–8.86)		1.00	
Secondary hospital	1,072 (5.13)	230 (12.05)	21.46 (19.03–24.04)		1.65 (1.37–1.98)	<0.001
Frequency of hospitalization				<0.001		
First-admitted	15,758 (75.40)	1,241 (65.04)	7.88 (7.46–8.31)		1.00	
Readmitted	5,141 (24.60)	667 (34.96)	12.97 (12.07–13.92)		1.24 (1.10–1.40)	<0.001
Length of hospitalization (days)				<0.001		
1–30	10,289 (49.23)	839 (43.97)	8.15 (7.63–8.70)		1.00	
31–60	7,584 (36.29)	712 (37.32)	9.39 (8.74–10.07)		0.97 (0.87–1.09)	0.606
≥61	3,026 (14.48)	357 (18.71)	11.80 (10.67–13.00)		0.95 (0.82–1.11)	0.531
Type of depression				<0.001		
Recurrent depressive disorder	10,092 (48.29)	1,067 (55.92)	10.57 (9.98–11.19)		1.00	
Depressive episode	10,516 (50.32)	831 (43.55)	7.90 (7.39–8.43)		1.01 (0.91–1.13)	0.821
Dysthymia	291 (1.39)	10 (0.52)	3.44 (1.66–6.23)		0.91 (0.46–1.80)	0.791
Hypertension				<0.001		
No	16,709 (79.95)	812 (42.56)	4.86 (4.54–5.20)		1.00	
Yes	4,190 (20.05)	1,096 (57.44)	26.16 (24.83–27.52)		3.01 (2.70–3.35)	<0.001
Hyperlipidemia				<0.001		
No	18,249 (87.32)	1,344 (70.44)	7.36 (6.99–7.75)		1.00	
Yes	2,650 (12.68)	564 (29.56)	21.28 (19.74–22.89)		1.69 (1.50–1.91)	<0.001
NAFLD				<0.001		
No	19,000 (90.91)	1,550 (81.24)	8.16 (7.77–8.56)		1.00	
Yes	1,899 (9.09)	358 (18.76)	18.85 (17.12–20.69)		1.58 (1.37–1.82)	<0.001
Lithium carbonate therapy				0.018		
No	20,453 (97.87)	1,882 (98.64)	9.20 (8.81–9.61)		1.00	
Yes	446 (2.13)	26 (1.36)	5.83 (3.84–8.43)		1.14 (0.73–1.78)	0.559
Antipsychotic drug				<0.001		
No	10,903 (52.17)	922 (48.32)	8.46 (7.94–8.99)		1.00	
Yes	9,996 (47.83)	986 (51.68)	9.86 (9.29–10.47)		1.04 (0.92–1.16)	0.530
Nootropic drug				0.259		
No	19,636 (93.96)	1,781 (93.34)	9.07 (8.67–9.48)		1.00	
Yes	1,263 (6.04)	127 (6.66)	10.06 (8.45–11.85)		0.81 (0.66–1.001)	0.051
Total	20,899 (100.00)	1,908 (100.00)	9.13 (8.74–9.52)			

### Prevalence of T2DM among depression inpatients

Among 20,899 depression inpatients, 1,908 inpatients had T2DM, and the prevalence of T2DM was 9.13% (95% CI, 8.74% to 9.52%). The annual prevalence of T2DM among depression inpatients is shown in Fig. 1, with the lowest prevalence in 2006 (2.47%) and the highest prevalence in 2015 (11.87%). From 2005 to 2018, the prevalence of T2DM showed an upward trend by year (trend χ^2^ = 54.48, *P* < 0.001). As shown in Table, the prevalence of T2DM showed no substantial difference in terms of sex, ethnicity, and nootropic drug use. The prevalence of T2DM increased with age (trend χ^2^ = 1,372.21, *P* < 0.001), from 0.39% in the 18 to 29 years group to 20.53% in the ≥70 years group. Compared with the married group (10.79%) and divorce/widowed/other group (15.18%), the prevalence of T2DM in the unmarried group was low (1.57%). In occupation subgroups, workers/farmers (10.26%) and retirees (21.78%) had relatively higher prevalence of T2DM. In medical insurance subgroups, patients with urban employee basic medical insurance (UEBMI) (12.39%) and urban resident basic medical insurance (URBMI) (13.98%) had a relatively higher prevalence of T2DM. In addition, the prevalence of T2DM was higher in patients who were readmitted (12.97%), hospitalized at secondary hospital (21.46%), and hospitalized for ≥61 d (11.80%). Depression patients with hypertension (26.16%), hyperlipidemia (21.28%), NAFLD (18.85%), and those receiving antipsychotic drugs (9.86%) had a higher prevalence of T2DM. Patients receiving lithium carbonate therapy had a lower prevalence of T2DM (5.83% versus 9.20%).

### Risk factors of T2DM among depression inpatients

The results of multivariate logistic regression analysis showed that T2DM was associated with age, occupation, medical insurance, hospital level, frequency of hospitalization, hypertension, hyperlipidemia, and NAFLD (Table). Specifically, all other older age groups had a higher risk of T2DM than the 18 to 29 years group (aOR ranged from 5.56 to 40.42). In terms of occupation, compared with the “manager of a government agency, public institution or enterprise”, the workers/farmers (aOR, 1.88; 95% CI, 1.24 to 2.84), retirees (aOR, 1.97; 95% CI, 1.33 to 2.92), “other occupation” group (aOR, 1.88; 95% CI, 1.25 to 2.83), and unemployed patients (aOR, 1.76; 95% CI, 1.13 to 2.73) had a higher risk of T2DM. Compared to patients with NCMS (new rural cooperative medical scheme), the patients with UEBMI (aOR, 1.33; 95% CI, 1.10 to 1.60) and out-of-pocket payment (aOR, 1.27; 95% CI, 1.04 to 1.56) had a higher risk of T2DM. In addition, risk of T2DM was higher in depression patients who were readmitted (aOR, 1.24; 95% CI, 1.10 to 1.40), hospitalized in secondary hospital (aOR, 1.65; 95% CI, 1.37 to 1.98), comorbid with hypertension (aOR, 3.01; 95% CI, 2.70 to 3.35), comorbid with hyperlipidemia (aOR, 1.69; 95% CI, 1.50 to 1.91), and comorbid with NAFLD (aOR, 1.58; 95% CI, 1.37 to 1.82).

### Stratified analysis

**Fig. 2.**
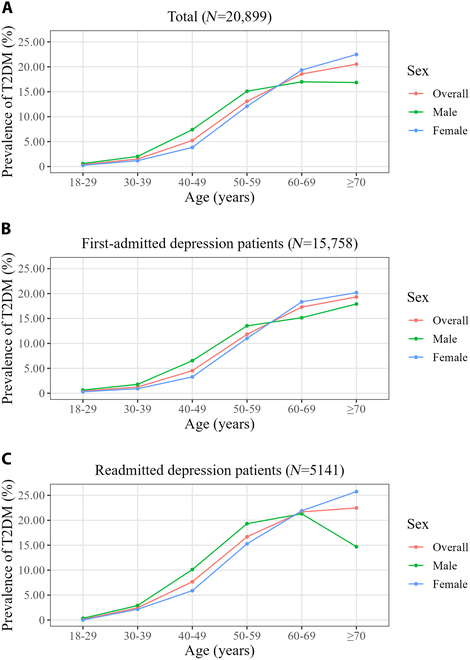
The prevalence of T2DM in different age groups stratified by sex. (A) Prevalence of T2DM among all depression patients. (B) Prevalence of T2DM among first-admitted depression patients. (C) Prevalence of T2DM among readmitted depression patients.

**Fig. 3.**
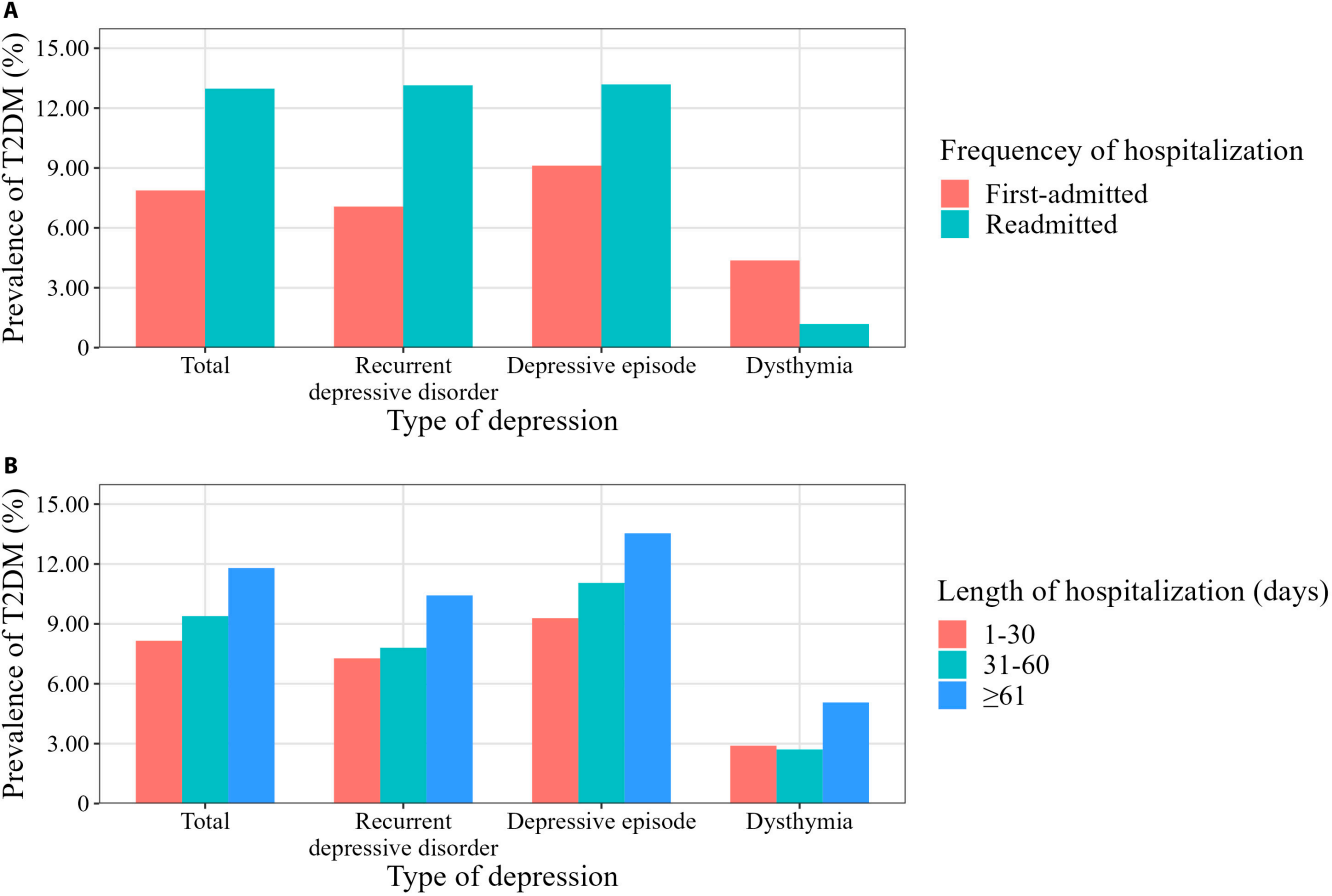
The prevalence of T2DM in different types of depression stratified by hospitalization status. (A) Prevalence of T2DM stratified by frequency of hospitalization. (B) Prevalence of T2DM stratified by length of hospitalization.

**Fig. 4.**
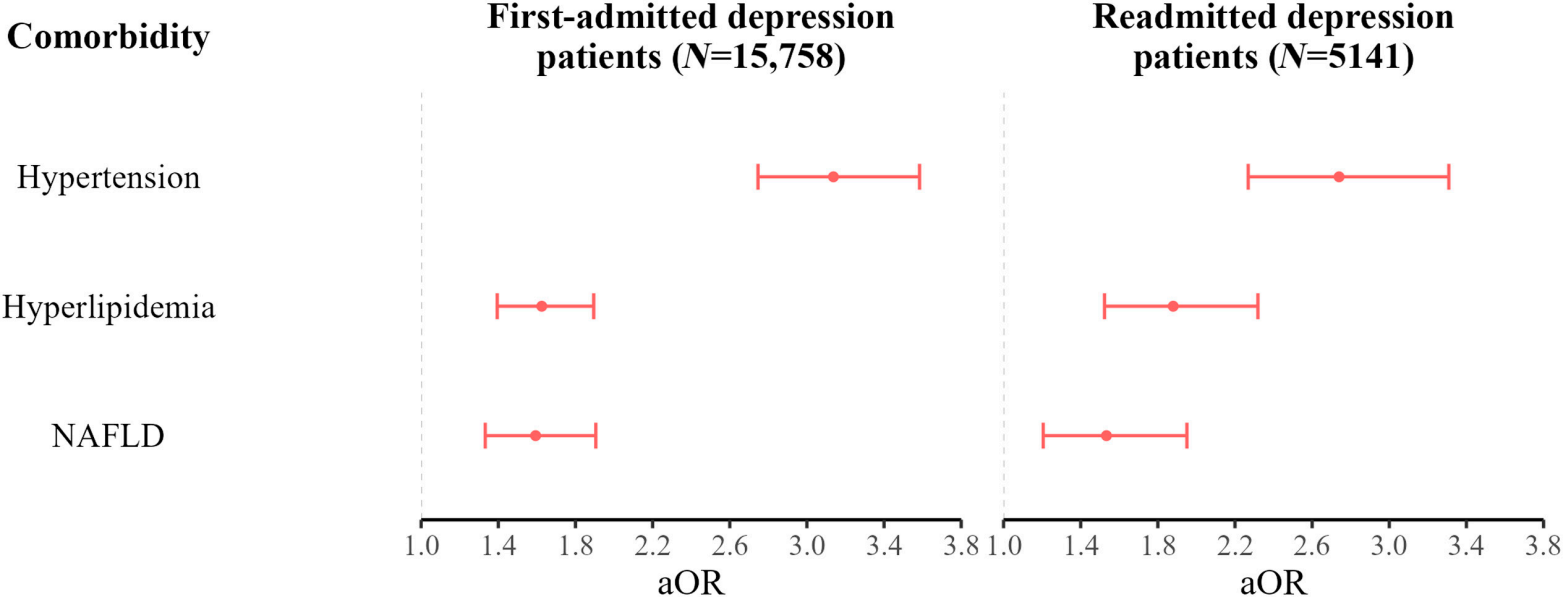
The association between T2DM and comorbidity stratified by frequency of hospitalization. Adjusted for sex, age, ethnicity, marital status, occupation, medical insurance, hospital level, length of hospitalization, type of depression, lithium carbonate therapy, antipsychotic drug use, and nootropic drug.

The results of the analysis stratified by age (18 to 39, 40 to 59, and ≥60 years) were summarized in Table S1. It was found that the hospital level, hypertension, hyperlipidemia, and NAFLD were associated with T2DM regardless of age group (Table S1). The association strength of hospital level (aORs decreased from 3.19 to 1.49), hypertension (aORs decreased from 5.15 to 2.75), hyperlipidemia (aORs decreased from 3.03 to 1.55), and NAFLD (aORs decreased from 2.62 to 1.41) with T2DM gradually decreased with age (Table S1). Among depression inpatients aged 40 to 59 years, the risk of T2DM was lower in females than in males (aOR, 0.80; 95% CI, 0.68 to 0.94) (Table S1). However, among depression inpatients ≥60 years old, the risk of T2DM was higher in females than in males (aOR, 1.18; 95% CI, 1.01 to 1.38) (Table S1). Further analysis showed that the prevalence of T2DM in men and women crossed over at the age of 60 (Fig. 2A). Specifically, the prevalence of T2DM in females was lower than in males among depression inpatients aged 18 to 59 years, while the prevalence of T2DM in females was higher than in males among depression inpatients aged ≥60 years. The same sex pattern was also found in the first-admitted (Fig. 2B) and readmitted (Fig. 2C) depression patients. Overall, the prevalence of T2DM had a similar pattern among patients with recurrent depressive disorder and depressive episode, and patients with dysthymia had a different pattern (Fig. 3). On the one hand, among patients with recurrent depressive disorder and depressive episode, the prevalence of T2DM was higher in those who were readmitted than those who were first-admitted, while it was the opposite among patients with dysthymia (Fig. 3A). On the other hand, among patients with recurrent depressive disorder and depressive episode, the prevalence of T2DM increased progressively in patients hospitalized for 1 to 30, 31 to 60, and ≥61 d, while this was not observed among patients with dysthymia (Fig. 3B). Regardless of the frequency of hospitalization, hypertension, hyperlipidemia, and NAFLD were associated with a higher risk of T2DM (Fig. 4).

## Discussion

At present, most studies examining the relationship between depression and diabetes have focused on patients with diabetes rather than depression. As far as we know, our study is one of few studies to comprehensively summarize the prevalence of T2DM in depression inpatients in China, and has a certain meaning for understanding the prevalence of diabetes in depression patients. Besides, depression patients were diagnosed by clinical criteria, and the results have better guidance for clinical work. In this study, it was found that the prevalence of T2DM among depression inpatients in Beijing was 9.13%, and showed an increasing trend from 2005 to 2018. There are differences in the prevalence of T2DM between subgroups of multiple variables. The multivariable logistic regression indicated that T2DM was associated with age, occupation, medical insurance, frequency and length of hospitalization, and comorbidities (hypertension, hyperlipidemia, and NAFLD). In stratified analysis, there was a different pattern of prevalence between males and females in different age groups (18 to 59 years versus ≥60 years).

During 2011–2012, the China Health and Retirement Longitudinal Study conducted among the population aged ≥45 years showed that the proportion of diabetes among depressive subjects was 12.8% [22]. This was lower than the population aged ≥50 years in our study. The prevalence of T2DM among major depressive disorder patients at a hospital in Belgium during 2002–2014 was 21.2% [23] and higher than the prevalence in our study. Although different diagnostic criteria have an impact on the prevalence of diabetes, the prevalence of diabetes in China increased in the past 3 decades for certain, and T2DM was the main contributor to the increase [24]. The prevalence of T2DM in this study also showed the same increase trend. A study also based on data collected at Beijing hospitals (which cover more than 80% of the population in Beijing) showed that the prevalence of T2DM in general patients increased from 3.7% in 2008 to 6.6% in 2017 [25]. Compared with general patients, depression inpatients have a higher prevalence of T2DM, which was 5.49% in 2008 and 7.27% in 2017%. This seems to support that depression increases the risk of T2DM. However, a multistage and stratified epidemiological survey conducted during 2015–2017 indicated that, in Beijing, the crude prevalence of diabetes was 17.0%, and the age- and sex-adjusted prevalence of diabetes was 13.6% [26]. The prevalence of T2DM among depression inpatients in any 1 year was lower than 13.6% and 17.0%. This may suggest that the risk of diabetes was not higher in patients with depression than in the general population. In fact, after summarizing studies on depression and diabetes, a review published in *The Lancet Diabetes & Endocrinology* indicated that depression might directly increase the risk of developing T2DM, and the associations between these 2 diseases were probably due to confounding factors [9]. It should be stated that comparisons between prevalence rates can only provide a reference and cannot conclude the relationship between depression and diabetes.

The result of multivariable logistic regression suggested that age is a risk factor for T2DM. In the national surveys for the Chinese conducted in 2013 and 2018, the prevalence of diabetes increased progressively with age [5], which is consistent with the age pattern in this study. The meta-analysis among Chinese showed that the risk of diabetes increased by 12% with each 1-year increase in age [27]. In our stratified analysis, the association strength of hypertension, hyperlipidemia, and NAFLD with T2DM gradually decreased with age, which suggested that age was an important risk factor of T2DM. In this study, workers/farmers, the “other occupations” group, retirees, and the unemployed had a higher risk of T2DM compared with managers. According to a WHO report, precarious employment (e.g., nonfixed term temporary contracts, being employed with no contract, and part-time work) and bad work conditions affect health [28]. Better employment, work, and socioeconomic conditions of managers may contribute to their lower risk of T2DM. A national study conducted for Swedish employees found that the incidence of T2DM was highest in manufacturing workers among men, and highest in manufacturing workers and cleaners among women [29]. In occupation subgroups, workers/farmers with depression had the second highest prevalence of T2DM after retirees with depression. The highest prevalence of T2DM among retirees with depression was mainly due to their older age rather than work conditions. The data from Swedish employed also showed that mortality rates were highest in manufacturing workers and machine operators among males with T2DM, and highest in manufacturing workers and cleaners among females with T2DM [30]. This suggests that more support should be given to depression patients of lower socioeconomic status, who not only have a higher prevalence of T2DM but also are more likely to suffer from health damage due to T2DM. In terms of medical insurance, patients with out-of-pocket payments had a higher risk of T2DM than patients with NCMS. This may be due to 2 reasons. On the one hand, patients with out-of-pocket payments tend to have lower socioeconomic status [31], which leads to a higher risk of T2DM. On the other hand, depression patients with out-of-pocket payments may not be able to afford other preventive health care because of the high financial burden of depression (depression patients spend about twice as much on health care as those without depression [32]). In addition, patients with UEBMI had a higher risk of T2DM than patients with NCMS. UEBMI and NCMS are Chinese government-led. The most obvious difference between these 2 medical insurances is the difference between urban and rural areas. Prevalence rates of T2DM among patients with URBMI (12.39%) and patients with UEBMI (13.98%) were similar, and they were both much higher than those among patients with NCMS (5.84%). This suggests that the prevalence of T2DM among depression patients had the same pattern as the prevalence of diabetes among the general population [5,26]. That is, the prevalence of T2DM among depression patients from urban areas was higher than those from rural areas. In China, tertiary hospitals have more beds and provide comprehensive medical services, while secondary hospitals have fewer beds and provide general medical services [20]. A higher risk of T2DM among depression patients hospitalized in secondary hospitals may result from less access to comprehensive care. It has been indicated that the prevalence of diabetes increases as depressive symptom severity worsens [33]. In our study, readmitted depression patients had a higher risk of T2DM, which may be due to their worse symptoms.

It was observed that depression with comorbid hypertension, hyperlipidemia, or NAFLD had a higher risk of T2DM. These associations remained significant in stratified analyses. The association between hypertension and diabetes may be related to arterial stiffness. Research has indicated that hyperglycemia and hyperinsulinemia (a compensatory rise in the early stage of diabetes) can induce vascular remodeling, resulting in heightened peripheral arterial resistance and the onset of hypertension [34]. In turn, arterial stiffness and impaired vasorelaxation contribute to the development of insulin resistance and diabetes [35]. For patients with hyperlipidemia, excessive triglyceride, cholesterol, and other lipid substances in the body are related to the occurrence and development of diabetes. High triglyceride level is widely recognized as a risk factor for T2DM. A recent cohort study has indicated that even increasing triglyceride levels within the normal range could increase the risk of developing T2DM [36]. In fact, the associations between the 3 comorbidities and diabetes are not unidirectional but rather involve a complex interconnection. For example, substantial evidence indicates that insulin resistance plays an important role in the occurrence and progression of essential hypertension, T2DM, and NAFLD [37,38]. This suggests that these diseases share a common physiological basis. Besides, some cross-sectional studies and longitudinal studies have shown that there is a connection between hypertension and NAFLD [39]. The concurrent presence of hypertension and NAFLD may be mediated by systemic inflammation, insulin resistance, and gut dysbiosis and potentially by oxidative stress, arterial stiffness, as well as genetic and epigenetic modifications [39]. In addition, excessive lipid substances not only cause hyperlipidemia but also increase the burden of liver metabolism and cause excessive lipid deposition in hepatocytes, which is an important pathophysiological process of NAFLD [37,40]. We suggest that future studies of the association between depression and T2DM should adjust for comorbidities in their models. Similar to the general population, depression inpatients in Beijing share some common risk factors for diabetes. An epidemiological survey conducted in 2014 among permanent residents in Beijing showed that older age, hypertension, and dyslipidemia were risk factors for diabetes, which was consistent with our study [41]. A national epidemiological survey conducted in China from 2015 to 2017 showed that older age, higher systolic blood pressure, higher triglyceride level, higher total cholesterol level, and higher low-density lipoprotein level were risk factors for diabetes, which was also consistent with our study [26]. However, these 2 epidemiological surveys lack some risk factors of our study, such as occupation, frequency of hospitalization, and nootropic drugs. This suggests that these variables may be specific risk factors for diabetes in depression inpatients. On the contrary, our study lacks some risk factors of these 2 epidemiological surveys, such as overweight, obesity, body mass index (BMI), heart rate, and use of alcohol. This suggests that these variables should be considered in the future study of diabetes among patients with depression.

Although some of the variables in this study were not associated with T2DM in the multivariate logistic regression analysis, the univariate chi-square test of these variables suggested that there were differences in the prevalence of T2DM between subgroups. In terms of marital status, unmarried depression inpatients had a low prevalence of T2DM. This may be related to the fact that unmarried subjects tend to be younger. A study conducted among Chinese suggested that depression symptoms were negatively associated with perceived social support from family, friends, and a significant other [42]. Divorced and widowed depression inpatients had the highest prevalence of T2DM, which may be due to their lower social support and worse depressive symptoms. As mentioned above, more severe depressive symptoms were associated with a higher prevalence of diabetes [33]. Some results may be explained by this association. First, the prevalence of T2DM increased progressively among depression patients hospitalized for 1 to 30, 31 to 60, and ≥61 d. Second, the prevalence of T2DM increased progressively among patients with dysthymia, depressive episode, and recurrent depressive disorder. Third, the prevalence of T2DM in depression treated with antipsychotic drugs was higher than that in those not treated with antipsychotic drugs (psychotic symptoms can occur in patients with severe depression). It may also be related to the fact that antipsychotics directly increase the risk of diabetes. Data from a national survey of people with psychosis suggested that antipsychotic drug treatment increased the risk of diabetes among patients who have no family history of diabetes [43]. In our study, the prevalence of T2DM was lower among depression receiving lithium carbonate therapy. However, like antipsychotic drugs, lithium carbonate showed no substantial results in multivariable logistic regression. A review showed that studies exploring the relationship between lithium and diabetes were few, with inconsistent results [44]. More studies about lithium carbonate therapy are needed in the future. In stratified analysis, a crossover was observed in the prevalence of T2DM between male and female depression inpatients at the age of 60 years. The increased prevalence of T2DM in women may be related to the decrease of estrogen after menopause. A national survey indicated that the younger the age of menopause, the higher the risk of T2DM [45]. A meta-analysis of randomized controlled trials suggested that postmenopausal hormone therapy reduces the levels of HbA1c and fasting glucose [46].

There are 3 limitations of this study. First, as mentioned in the previous discussion, the prevalence of T2DM in both the general population and the depressed population shows an increasing trend over time, and the prevalence varies from year to year. Therefore, the prevalence of T2DM in our study is an average from 2005 to 2018. Second, this study was cross-sectional; the causality between depression and T2DM cannot be determined. Finally, 2 variables that may have an impact on the logistic model were missing from the medical record front page data: BMI and the use of antidepressant medication.

## Conclusion

In summary, the prevalence of T2DM among depression inpatients from 2005 to 2018 in Beijing was 9.13% and showed an upward trend with the year. Depression patients who were older and with hypertension, hyperlipidemia, and NAFLD had a higher prevalence and higher risk of T2DM. Compared with male depression inpatients, female depression inpatients aged over 60 with depression had a higher prevalence and risk of T2DM. Psychiatrists should pay more attention to these high-risk groups of T2DM while treating depression.

## Ethical Approval

The study used information that is available in the database of the Beijing Municipal Commission of Health and Family Planning Information Center, and all identifiable information was removed.

## Data Availability

Data may be obtained from a third party and are not publicly available. For access to the data, please try to contact the corresponding author.
