# Supplementary material for: Prevalence and Risk Factors of Type 2 Diabetes Mellitus among Depression Inpatients from 2005 to 2018 in Beijing, China
Source: Health Data Sci. 2025 Mar 5;5:0111. doi: 10.34133/hds.0111 (PMC11880573; doi:10.34133/hds.0111)
Supplement: Supplementary 1 — Table S1 [file hds.0111.f1.docx]

Table S1. Prevalence and risk factors of T2DM among depression inpatients by age during 2005-2018

| **Characteristic** | **18-39 years** | | | |  | **40-59 years** | | | |  | **≥60 years** | | | |
| --- | --- | --- | --- | --- | --- | --- | --- | --- | --- | --- | --- | --- | --- | --- |
|  | **Prevalence of T2DM (%)** | ***P* for fisher’s exact test*** | **aOR (95% CI)** | ***P* value for aOR** |  | **Prevalence of T2DM (%)** | ***P* for chi-square test** | **aOR (95% CI)** | ***P* value for aOR** |  | **Prevalence of T2DM (%)** | ***P* for chi-square test** | **aOR (95% CI)** | ***P* value for aOR** |
| **Sex** |  | 0.026 |  |  |  |  | <0.001 |  |  |  |  | 0.002 |  |  |
| Male | 1.14 |  | 1.00 |  |  | 11.42 |  | 1.00 |  |  | 16.94 |  | 1.00 |  |
| Female | 0.63 |  | 0.75 (0.43, 1.31) | 0.316 |  | 8.70 |  | 0.80 (0.68, 0.94) | 0.006 |  | 20.53 |  | 1.18 (1.01, 1.38) | 0.033 |
| **Ethnicity** |  | 0.498 |  |  |  |  | 0.848 |  |  |  |  | 0.129 |  |  |
| Han Chinese | 0.83 |  | 1.00 |  |  | 9.71 |  | 1.00 |  |  | 19.29 |  | 1.00 |  |
| Non-Han Chinese | 1.21 |  | 1.93 (0.73, 5.12) | 0.184 |  | 9.14 |  | 1.11 (0.75, 1.63) | 0.607 |  | 22.50 |  | 1.30 (0.91, 1.85) | 0.150 |
| Unknown | 0.00 |  | — | — |  | 8.11 |  | 0.89 (0.37, 2.14) | 0.799 |  | 11.43 |  | 0.64 (0.30, 1.37) | 0.254 |
| **Marital status** |  | 0.049 |  |  |  |  | 0.839 |  |  |  |  | <0.001 |  |  |
| Married | 1.01 |  | 1.00 |  |  | 9.73 |  | 1.00 |  |  | 18.41 |  | 1.00 |  |
| Unmarried | 0.67 |  | 0.93 (0.52, 1.65) | 0.802 |  | 8.79 |  | 0.75 (0.49, 1.15) | 0.190 |  | 20.00 |  | 1.02 (0.56, 1.85) | 0.951 |
| Divorced/Widowed/Other | 1.87 |  | 1.77 (0.60, 5.20) | 0.297 |  | 9.47 |  | 0.90 (0.67, 1.19) | 0.460 |  | 24.41 |  | 1.16 (0.96, 1.41) | 0.129 |
| **Occupation** |  | <0.001 |  |  |  |  | <0.001 |  |  |  |  | <0.001 |  |  |
| Manager of government agency, public institution or enterprise | 0.00 |  | — | — |  | 4.98 |  | 1.00 |  |  | 4.35 |  | 1.00 |  |
| Civil servant/Professional/Office clerk | 0.82 |  | 1.00 |  |  | 7.19 |  | 0.95 (0.56, 1.60) | 0.834 |  | 13.53 |  | 2.35 (1.004, 5.52) | 0.049 |
| Worker/Farmer | 1.41 |  | 2.50 (0.81, 7.75) | 0.112 |  | 10.90 |  | 1.32 (0.79, 2.19) | 0.285 |  | 16.48 |  | 2.96 (1.39, 6.31) | 0.005 |
| Other | 0.75 |  | 1.31 (0.56, 3.07) | 0.530 |  | 9.84 |  | 1.48 (0.93, 2.36) | 0.098 |  | 18.64 |  | 3.29 (1.59, 6.82) | 0.001 |
| Retiree | 60.00 |  | 60.41 (6.19, 589.53) | <0.001 |  | 13.71 |  | 1.44 (0.85, 2.45) | 0.180 |  | 25.38 |  | 3.46 (1.65, 7.26) | <0.001 |
| Unemployed | 1.00 |  | 1.27 (0.43, 3.70) | 0.665 |  | 9.62 |  | 1.17 (0.68, 2.01) | 0.561 |  | 21.30 |  | 3.25 (1.45, 7.25) | 0.004 |
| **Medical insurance** |  | 0.001 |  |  |  |  | <0.001 |  |  |  |  | <0.001 |  |  |
| NCMS | 0.42 |  | 1.00 |  |  | 7.76 |  | 1.00 |  |  | 13.77 |  | 1.00 |  |
| UEBMI | 1.69 |  | 2.96 (1.07, 8.23) | 0.037 |  | 11.01 |  | 1.22 (0.93, 1.59) | 0.144 |  | 21.86 |  | 1.32 (0.995,1.75) | 0.054 |
| URBMI | 0.50 |  | 0.73 (0.07, 7.41) | 0.791 |  | 14.04 |  | 1.29 (0.84, 1.98) | 0.249 |  | 22.92 |  | 1.16 (0.79, 1.71) | 0.439 |
| Free medical service | 0.00 |  | — | — |  | 9.79 |  | 1.28 (0.74, 2.20) | 0.379 |  | 19.25 |  | 1.22 (0.75, 1.99) | 0.411 |
| Other insurance | 0.42 |  | 1.25 (0.36, 4.32) | 0.721 |  | 6.72 |  | 0.95 (0.70, 1.30) | 0.762 |  | 13.94 |  | 1.06 (0.77, 1.46) | 0.731 |
| Out-of-pocket | 0.81 |  | 1.83 (0.65, 5.17) | 0.253 |  | 10.27 |  | 1.23 (0.93, 1.65) | 0.150 |  | 20.25 |  | 1.25 (0.92, 1.71) | 0.155 |
| **Hospital level** |  | 0.015 |  |  |  |  | <0.001 |  |  |  |  | <0.001 |  |  |
| Tertiary hospital | 0.79 |  | 1.00 |  |  | 9.15 |  | 1.00 |  |  | 18.32 |  | 1.00 |  |
| Secondary hospital | 2.89 |  | 3.19 (1.03, 9.86) | 0.044 |  | 19.90 |  | 2.06 (1.52, 2.79) | <0.001 |  | 29.08 |  | 1.49 (1.18, 1.89) | 0.001 |
| **Frequency of hospitalization** |  | 0.188 |  |  |  |  | <0.001 |  |  |  |  | <0.001 |  |  |
| First-admitted | 0.77 |  | 1.00 |  |  | 8.58 |  | 100 |  |  | 18.00 |  | 1.00 |  |
| Readmitted | 1.16 |  | 1.17 (0.63, 2.18) | 0.628 |  | 13.08 |  | 1.46 (1.22, 1.74) | <0.001 |  | 22.04 |  | 1.12 (0.95, 1.31) | 0.166 |
| **Length of hospitalization (days)** |  | 0.704 |  |  |  |  | 0.730 |  |  |  |  | 0.150 |  |  |
| 1-30 | 0.91 |  | 1.00 |  |  | 9.53 |  | 1.00 |  |  | 18.95 |  | 1.00 |  |
| 31-60 | 0.71 |  | 0.81 (0.45, 1.48) | 0.493 |  | 9.61 |  | 1.02 (0.86, 1.21) | 0.803 |  | 18.64 |  | 0.98 (0.83, 1.15) | 0.759 |
| ≥61 | 0.88 |  | 0.61 (0.23, 1.57) | 0.303 |  | 10.31 |  | 0.92 (0.72, 1.18) | 0.494 |  | 21.38 |  | 1.03 (0.84, 1.26) | 0.770 |
| **Type of depression** |  | 0.243 |  |  |  |  | 0.180 |  |  |  |  | 0.144 |  |  |
| Recurrent depressive disorder | 1.06 |  | 1.00 |  |  | 10.25 |  | 1.00 |  |  | 19.95 |  | 1.00 |  |
| Depressive episode | 0.71 |  | 0.78 (0.45, 1.35) | 0.375 |  | 9.07 |  | 1.04 (0.89, 1.22) | 0.611 |  | 18.58 |  | 0.99 (0.85, 1.15) | 0.891 |
| Dysthymia | 0.49 |  | 0.56 (0.07, 4.45) | 0.580 |  | 11.29 |  | 1.26 (0.55, 2.86) | 0.585 |  | 7.69 |  | 0.39 (0.09, 1.72) | 0.215 |
| **Hypertension** |  | <0.001 |  |  |  |  | <0.001 |  |  |  |  | <0.001 |  |  |
| No | 0.70 |  | 1.00 |  |  | 6.34 |  | 1.00 |  |  | 11.33 |  | 1.00 |  |
| Yes | 8.21 |  | 5.15 (2.32, 11.43) | <0.001 |  | 22.69 |  | 3.53 (3.02, 4.14) | <0.001 |  | 29.59 |  | 2.75 (2.38, 3.19) | <0.001 |
| **Hyperlipidemia** |  | <0.001 |  |  |  |  | <0.001 |  |  |  |  | <0.001 |  |  |
| No | 0.66 |  | 1.00 |  |  | 8.31 |  | 1.00 |  |  | 16.51 |  | 1.00 |  |
| Yes | 4.13 |  | 3.03 (1.50, 6.11) | 0.002 |  | 18.47 |  | 1.75 (1.44, 2.12) | <0.001 |  | 29.64 |  | 1.55 (1.32, 1.83) | <0.001 |
| **NAFLD** |  | <0.001 |  |  |  |  | <0.001 |  |  |  |  | <0.001 |  |  |
| No | 0.63 |  | 1.00 |  |  | 8.58 |  | 1.00 |  |  | 17.99 |  | 1.00 |  |
| Yes | 4.12 |  | 2.62 (1.32, 5.19) | 0.006 |  | 18.53 |  | 1.60 (1.30, 1.96) | <0.001 |  | 30.84 |  | 1.41 (1.14, 1.74) | 0.001 |
| **Lithium carbonate therapy** |  | 0.737 |  |  |  |  | 0.223 |  |  |  |  | 1.000 |  |  |
| No | 0.83 |  | 1.00 |  |  | 9.62 |  | 1.00 |  |  | 19.32 |  | 1.00 |  |
| Yes | 1.03 |  | 1.03 (0.30, 3.54) | 0.965 |  | 13.51 |  | 1.25 (0.70, 2.25) | 0.459 |  | 18.60 |  | 0.88 (0.40, 1.96) | 0.758 |
| **Antipsychotic drug** |  | 0.442 |  |  |  |  | <0.001 |  |  |  |  | 0.001 |  |  |
| No | 0.75 |  | 1.00 |  |  | 8.63 |  | 1.00 |  |  | 17.68 |  | 1.00 |  |
| Yes | 0.93 |  | 0.98 (0.54, 1.78) | 0.942 |  | 10.87 |  | 1.11 (0.94, 1.32) | 0.232 |  | 21.18 |  | 0.998 (0.851, 1.172) | 0.983 |
| **Nootropic drug** |  | 1.000 |  |  |  |  | 0.146 |  |  |  |  | 0.354 |  |  |
| No | 0.84 |  | 1.00 |  |  | 9.80 |  | 1.00 |  |  | 19.16 |  | 1.00 |  |
| Yes | 0.83 |  | 1.03 (0.30, 3.48) | 0.965 |  | 7.69 |  | 0.62 (0.44, 0.89) | 0.007 |  | 21.18 |  | 0.97 (0.74,1.26) | 0.796 |
| **Total** | 0.84 |  |  |  |  | 9.67 |  |  |  |  | 19.31 |  |  |  |

Note: * Among depression inpatients aged 18-39 years, the number of T2DM cases was small, and the "fisher’s exact test" was more appropriate to compare the differences in prevalence between subgroups. T2DM= type 2 diabetes mellitus; aOR=adjusted odds ratio; NCMS=new rural cooperative medical scheme; UEBMI=urban employee basic medical insurance; URBMI=urban resident medical insurance; NAFLD=nonalcoholic fatty liver disease.
